# Trainees are human too

**DOI:** 10.1177/0036933020983567

**Published:** 2020-12-25

**Authors:** Eliot L Rees

**Affiliations:** 1 Lecturer in Medical Education, School of Medicine, Keele University, UK; 2 NIHR Academic Clinical Fellow in General Practice, Research Department of Primary Care and Population Health, University College London, UK

Dear Editor,

I read with interest the article by Laloo et al.^1^ and commend them on their efforts to preserve educational provision for core surgical trainees. However, I was concerned with their statement that “as the study involved trainees and not patients, ethical approval was not necessary”.^1^

The declaration of Helsinki states that research involving human subjects should be reviewed by an ethics committee. I urge authors, reviewers, and editors to remember that while students and trainees are not patients, they are still human. Consequently, if they are involved as participants in research studies that research should have had received either a favourable opinion or exemption from a research ethics committee.

That is not to say that all manuscripts including data collected from learners require ethical approval. It is widely accepted that evaluation reports are exempt. However, the lines between educational evaluation and medical education research are sometimes blurred. Guidance from the editors of *The Clinical Teacher* may be useful in distinguishing between the two.^2^ According to their definitions, Laloo’s article would be considered evaluation. Still, it is important to ensure there was informed consent, and an expedited review or exemption from a research ethics committee should have been obtained.^3^

I recognise the difficulty inherent in suggesting all educational evaluations should be reviewed by a research ethics committee. Schuwirth and Durning argue that the effort expended on the preparation and review of ethics proposal can be disproportionate to the risk posed.^4^ This concern is particularly appropriate at present when there is an imperative to disseminate educational innovations in response to the disruption caused by the COVID-19 pandemic. Nevertheless, we have a duty to our learners to ensure that research and evaluation on these innovations are conducted and reported in an ethical manner.
